# Neurogenetics of Brain Connectivity: Current Approaches to the Study (Review)

**DOI:** 10.17691/stm2024.16.1.07

**Published:** 2024-02-28

**Authors:** E.A. Proshina, T.S. Deynekina, O.V. Martynova

**Affiliations:** Researcher, Centre for Cognition & Decision Making, Institute for Cognitive Neurosciences; National Research University Higher School of Economics, 20 Myasnitskaya St., Moscow, 101000, Russia; Analyst; Center for Strategic Planning and Management of Biomedical Health Risks of the Federal Medical Biological Agency, 10 Pogodinskaya St., Moscow, 119121, Russia; Deputy Director, Head of the Laboratory of Human Higher Nervous Activity; Institute of Higher Nervous Activity and Neurophysiology, Russian Academy of Sciences, 5A Butlerova St., Moscow, 117485, Russia, Associate Professor, Department of Biology and Biotechnology; National Research University Higher School of Economics, 20 Myasnitskaya St., Moscow, 101000, Russia

**Keywords:** connectivity, fMRI, EEG, genome-wide association study, heritability, neural network, neurogenetics

## Abstract

Owing to the advances of neuroimaging techniques, a number of functional brain networks associated both with specific functions and the state of relative inactivity has been distinguished. A sufficient bulk of information has been accumulated on changes in connectivity (links between brain regions) in psychopathologies, for example, depression, schizophrenia, autism. Their genetic markers are being actively investigated using a candidate-gene approach or a genome-wide association study. At the same time, there is not much data considering connectivity as an intermediate link in the genotype–pathology chain, although it seems to be a reliable endophenotype, since it demonstrates a high stability and high heritability. In the present review, we consider the results of investigations devoted to the search for biomarkers, molecular and genetic associations of functional, partially anatomical, and effective connectivity. The main approaches to the evaluation of connectivity neurogenetics have been described, as well as specific genetic variants, for which the association with brain connectivity in psychiatric pathologies has been detected.

## Introduction

Assessment of brain regions activity synchronization makes it possible to distinguish sufficiently stable networks of functionally connected regions associated with spontaneous brain activity and with a goal-directed behavior. The assumption of the existence of a specific genetic substance related to functional connectivity has been confirmed by the recent studies. Associations have been found between the parameters of the brain structures determined with various functional diagnostic methods and genetic polymorphism and/or gene expression. Taking into account a wide spread of brain connectivity assessment techniques, it may be supposed that in the nearest future, patterns of connectivity may be used in diagnostic purposes in clinical practice. Besides, sufficient information has been accumulated on the links between brain regions in psychopathologies. There are multiple studies aimed at the detection of a genetic background of psychiatric diseases. However, data considering connectivity as an intermediate link in the genotype–pathology chain is not plentiful, although it seems to be a reliable endophenotype, since it shows a high stability and high heritability.

The present review considers the most important concepts, methods, and theories, and informs about the most common genetic markers of alterations in functional connectivity in psychopathologies encountered in the literature. Since the genome-wide association study method is increasingly being used and there is a broad diversity of candidate genes explored in relation to brain connectivity and psychiatric/neurological disorders, it is not possible to cover all the problems within the frameworks of a single review. However, the analyzed material will help the specialists from different fields of medicine, psychology, neurosciences, who are not engaged in searching for genetic markers, to get an idea about this area of investigations.

The PubMed, eLIBRARY.RU databases and Google Scholar search engine were used in the process of our work. Searching was performed using the following key words and their combinations: “brain connectivity”, “genetic marker”, “genetics”, “genome-wide association study”, “polymorphisms” (without reference to the year of publication). Papers published from 1989 to 2022 were included into the review. All in all, 90 works have been examined.

## Materials and Methods

***The aim of this work*** was to review the investigations devoted to the search for biomarkers, molecular and genetic associations of functional (and partially anatomical and effective) connectivity.

### The structure of the article

*Part 1* is devoted to the analysis of the current approaches to the estimation of human brain connectivity and instrumental methods used for this aim. Reasons are given, why the assessment of brain connectivity is a promising potential marker of psychiatric diseases.

*Part 2* contains information on the contribution of heritability to the patterns of structural and functional connectivity.

*Part 3* considers molecular genetic markers of functional brain connectivity.

*Part 4* overviews the studies of genetic associations of brain connectivity in pathology.

## 1. Brain connectivity as an endophenotype

As the amount of neurons is enormous and the number of synaptic contacts is still much greater, the complexity of brain communicative structure at the tissue level defies currently formal description [1]. Rapid development of neuroimaging techniques in the early twenty first century initiated the study of functions of not only separate brain regions but the anatomical and functional networks as well. Anatomical links of the brain regions provided by the white matter tracts are called structural (anatomical) connectivity. Anatomical connectivity may be assessed by diffuse tensor imaging using MRI (DT-MRI). The term “functional connectivity” denotes the activity of definite brain regions synchronized in time in the process of performing a specific task or in the resting state [2, 3]. Functional connectivity is supposed to mediate the information processing in the brain.

When studying structural and functional connectivity, it has been found that links between the regions of interest typical for fulfilling cognitive/behavioral functions, reflect more likely statistical relationships and not always correspond to anatomical connections [4]. Although functional connections in a resting state are changeable and are present between the regions without structural connection, their strength, stability, and spatial statistics are, nevertheless, limited by a large-scale anatomical structure of the human cerebral cortex [5]. For the first time functional connectivity was examined using functional MRI (fMRI), possessing high spatial resolution (up to 1 mm^3^) [6], and positron emission tomography (PET). Presently, the development of analytical methods allows visualization of functional networks based on the EEG data both at the levels of a sensor and cortical generators.

In addition to structural and functional connectivity, there is effective connectivity, suggesting causal relationships between the brain regions in the process of performing specific functions [7]. A high temporal resolution of electro- and magnetoencephalography and the fact that their signals depend on electrophysiological activity of neurons determine the priority of these methods in estimating effective connectivity, however, conclusions must be interpreted keeping in mind existing constrictions.

Data on the number of networks functioning in the human brain are controversial, however, the following networks have been well-established: default mode networks (DMN), the only network, whose activity increases in the absence of purposive activity; and the networks positively connected with the tasks: visual network (VN); somatomotor network (SMN); dorsal attention network (DAN); ventral attention network (VAN); fronto-parietal control network (FPN) or central executive network; limbic network (LN); saliency network (SN) [8, 9]. The detailed topological analysis allows the selection of 15–20 neural networks whose activity is associated with motor control, perception of sensory information, and episodic memory, spatial orientation, implementation of verbal functions [10]. Neural networks are interconnected, and the sets of the regions involved in them may partially overlap. A certain level of hierarchy between the networks maintains realization of functions.

Functional networks are a convenient object for exploration since they can be analyzed with a great variety of mathematical tools. In addition to determining the strength of connections between the brain regions using the analysis of phase-frequency characteristics, one of a widely used approach is a graph analysis, which allows the selection of metrics characterizing the network architecture.

In the current research studies, brain connectivity and graph metrics are suggested to be endophenotypes during investigations of associations between genes and clinical symptoms or behavioral manifestations. The literature data confirm that exploration of associations of a candidate gene with brain activity patterns (using neuroimaging) is an easier task than studying a direct effect of a gene on the behavioral phenotype [11]. Besides, it should be taken into consideration that clinical symptoms are a final manifestation of the disease process, therefore, data on functional connectivity and the identified genetic associations may serve as a valuable material for timely detection of pathology. Searching for patterns of connectivity and its genetic markers is of practical use for translational medicine and for studying the mechanisms of variability of personal and situational behavior in norm and in pathology [12–15]. It has been found that psychopathological states are often characterized by changes in functional connectivity [12–14].

Thus, considering connectivity as an endophenotype represents implementation of the systemic approach in neurobiology and provides the possibility of studying the effect on behavior/clinical symptoms of a genotype mediated by specific patterns of activity/anatomy of the brain.

## 2. Heritability of structural and functional connectivity

At first, to assess the influence of genetic factors, emphasis was placed on the exploration of structural connectivity. The analysis of genetic correlations and quantitative trait loci (QTL) pointed to the hereditable nature of fractional anisotropy reflecting the directions of axonal tracts. The linkage of fraction anisotropy and radial diffusivity with region D15S816 on chromosome 15q25 and region D3S1754 on chromosome 3q27, respectively, has also been demonstrated [16]. It should be noted that these loci were associated with mental disorders such as depression and obsessivecompulsive disorder. A significant link of the regions of the chromosome 15q22-q23 and anatomical connectivity has been shown [16].

The investigation, combining DT-MRI and genomewide association study (GWAS), performed on a large sample of monozygotic and dizygotic twins, has shown association of polymorphism in genes *dlgap2* and *spon1* with structural connectivity in the cortex [17]. The proteins Spondin 1 (SPON1) and DLG associated protein 2 (DLGAP2) translated by these genes are involved in the processes of intercellular matrix structuring and cell adhesion, influencing intercellular contacts during the implementation of structural connections. Besides, there have been found significant associations of structural connectivity and structural integrity of the white matter with polymorphism of the genes of brain-derived neurotrophic factor (BDNF), NTRK1-receptor for nerve growth factor (NGF), catechol-o-methyltrasferase (COMT), clusterin (CLU), ErbB-2 receptor tyrosine kinase 4 (ErbB-4), homeostatic iron regulator (HFE) [18].

The comparison of data on a heritable polygenic nature of schizophrenia and autism and on intensive disorder of structural connectivity of the cerebral cortex in these diseases [19, 20] encouraged us to think that functional connectivity may also have genetic correlates [21–23]. A general scoring of heritability of functional connectome h2 is 0.42–0.60, which demonstrates the contribution of genetic factors to the formation of connectome endophenotype [21, 24, 25].

The majority of studies in the field of connectome neurogenetics apply graph theory and evaluate the effects of genes exactly in relation to the metrics of neural networks [18, 26]. Graph analysis allows for computing the metrics such as characteristic path length, clustering coefficient, modularity, global efficiency, small-world structure, which are interpreted in terms of information transfer efficiency in the network [27–30]. Studies of monozygotic and dizygotic twins have shown that in various EEG frequency ranges, 46–89% of individual differences in the clustering coefficient and 37–62% of individual differences in the characteristic path length are inheritable. This allows us to express assumptions that graph metrics are promising markers of genetic differences in brain functioning [31].

The studies based on graph analysis have found that the efficiency of the rich club modules (brain regions possessing the greatest amount of connections) and a global architecture of networks are most sensitive to polymorphism or gene expression [32], in particular, genes, encoding proteins, which provide reactions of central metabolism and implementation of electrophysiological properties of neurons. This assumption is consistent with the results of systemic analysis, which indicate a high heritability of global connectivity in comparison with local [33].

It has been found that during the performance of a task involving working memory, the brain of the healthy participants corresponds to the structure of the “small world” in the alpha, beta, and gamma ranges of the EEG, whereas in the group with schizophrenia no such correspondence was observed, suggesting disorders in the organization of neural networks in patients with schizophrenia [34].

Investigations of functional networks using EEG are often focused on the study of spontaneous brain activity, since high heritability of human resting-state EEG characteristics has been confirmed [35]. Functional MRI (fMRI) has shown that individual spontaneous neuron activity in the resting state correlates with the activity of functional networks involved in the cognitive tasks, suggesting that resting-state activity patterns can be studied as endophenotypes of cognitive abilities [36, 37].

## 3. Approaches to identifying molecular genetic markers of functional brain connectivity

Connectome neurogenetics is assessed in four experimental paradigms of associative research. The following associations are estimated: candidate phenotype–candidate gene association (CP–CGA), or candidate phenotype–genome-wide association (CP–GWA), or association between any changes in neuroimaging data and gene (at the level of a voxel or the region of interest), i.e. brain–wide, candidate gene association (BW–CGA), or between any changes in the neuroimaging data and multigenome data, i.e. brain-wide–genome-wide association (BW–GWA) [38].

A specific candidate gene or a single nucleotide polymorphism (SNP) may be chosen as a molecular genetic parameter, or GWAS data may also be used. However, in the second case, the analysis can be carried out using data of lower dimensionality compared to the initial set of parameters [39].

Thus, SNPs may be ranked and grouped using various approaches, for example, clustering based on the analysis of functional identity [40–42], transforming in this way a set of univariate data to multivariate. Considering the specificity of biological samples, public data sources are usually used for brain transcriptome analysis, such as data from the Allen Human Brain Atlas (AHBA) or the UK Biobank [43, 44].

Imaging data of neural tissue activity may be analyzed using a voxel-wise approach for the whole brain volume [45]. In the visualization domain, voxel is a minimally possible estimated volume of a 3D image. While this approach has the potential to identify new significant associations, the high granularity of the data makes it impossible to assess the connections between separate cortical regions. Besides, testing statistical significance of associations requires sufficient computation power to make multiple comparisons (in case of BW–GWA it is about 10^10^). The initial high visualization dimensionality of data may be reduced if associations are analyzed in relation to the regions of interest, which are determined either according to a certain parcellation algorithm (seed-based approach) [46], or within the frameworks of the existing researchers’ hypothesis.

It should be noted that, in this case, conceptual imperfection of the approach to the estimation of interconnection between the pattern of connectivity and genetic markers is caused by variability in the methods of brain region parcellation, i.e. segregation of the modules, which are integrated into networks, and also by interindividual variability and dynamism of these functionally delineated regions [47–49]. Therefore, for better comparability and reproducibility of the results, delineation of the brain regions, which are the regions of interest of the main neural networks such as DMN, dorsal attention network, and others, is often used as a scheme of parcellation. Definitions are also made using functional atlases of the parcellated regions on the basis of the regional functional connectivity on a large volume of fMRI data obtained from healthy volunteers [50].

## 4. Investigations in the genotype–connectivity– phenotype paradigm

A large pool of researches is devoted to the effect of candidate genes on brain connectivity in schizophrenia. Zhang et al. [51] evaluated the effect of the *nrgn* gene (rs12807809) polymorphism on the connectivity of the hippocampal formation in 59 patients with schizophrenia and 99 healthy subjects. Neurogranin (Nrgn) protein encodes the substrate of postsynaptic protein kinase C, binding to calmodulin in the absence of calcium, and polymorphism of its gene is associated with schizophrenia. Changes in functional connectivity (measured as the correlation of activities in the hippocampus and cortex or subcortical structures) in schizophrenia have been found to be associated with polymorphism of the *nrgn* gene.

The role of the *COMT* gene polymorphism is well known in relation to schizophrenia [52, 53], including determination of cognitive impairment level [54]. The val108/158met polymorphism is significantly associated with the reduction of connectivity in the cortex of healthy individuals (n=22). At the same time, the level of cognitive functions, measured by diverse tests, did not differ in the carriers of various alleles (n=496–1218) [55].

Using fMRI images of 800 healthy individuals and the GWAS method in their later study, Zhang et al. [42] separated 90 cortical regions and 7 resting-state neural networks and analyzed associations with the levels of local gene expression. The results have shown that the majority of significant functional connections between the regions (78%) referred to the pool of interaction within the brain neural networks, while a share of internetwork connectivity was 22% of the total number of connections. 125 genes, whose expression was significantly associated with connectivity, referred to the clusters of dendrite growth regulation, response to the external stimulus and signaling pathways of metabotropic receptors, secretion and transport of the protein, regulation of the intracellular calcium pool (among the newly identified were genes *mcub*, *doc2b*, *pipor2*, *adtrp*, *ifnlr1*, *pmepa1*). Expression of 51 genes was common for connectivity of all considered neural networks. All these genes referred to the cluster of regulation of action potential, ion transport and homeostasis, metabotropic receptor signaling, energy metabolism, and immune response. It is notable that 17 of 51 and 34 of 125 detected genes were associated specifically with schizophrenia rather than with other disorders (for example, such as autism spectrum disorder, attention deficit hyperactivity disorder, major depressive disorder). This speaks in favor of the fact that impairment of functional connectivity is the most prominent neuroimaging marker of schizophrenia.

In this regard, interesting are investigations of modular playotropy — attempts to determine interrelationships between the sets of phenotypes and the sets of genes specifying them relative to the network architecture of the human brain [56, 57]. It has been shown that for each region of interest, an almost unique set of SNPs and gene transcription profiles correspond to the connectivity pattern [58]. The metadata analysis pointed to a certain functional isolation of the SNPs clusters. There were significant associations of the genes of iron transport and storage proteins (connected by magnetic susceptibility of the brain subcortical tissue); extracellular matrix and epidermal growth factor (connected with the white matter microstructure); regulation of the midline axon development, signal transmission, and plasticity with SNP [59]. The detected results regarding gene expression, SNPs, and human behavior may help in searching for candidate genes of psychiatric diseases, because if the brain regions associated with the disease are known, it is likely that single-nucleotide polymorphisms in the genes, whose coexpression reflects the connectivity of these regions, are involved in the pathogenesis of this disease [58].

Besides, investigations of SNPs, gene transcript profiles, and metrics of structural and functional connectivity in the process of performing behavioral tests have shown the significance of associations specifically in relation to functional connectivity. This points to the fact, that genetic determinacy of some behavioral reactions is realized exactly at the level of functional connectivity [58]. It was similarly demonstrated in a series of other studies that it is in respect to the dynamics of functional connectivity that the gene effect is so marked [60, 61]. Apart from this, the comparison of data shows that despite the differences in the patterns of functional connectivity in the studies using EEG and fMRI, dynamic changes of such connectivity during cognitive tasks (watching video, flashing gratings) appear to be consistent among the individuals regardless of the method of recording brain activity [62].

When studying neurodegenerative diseases, it has been found that allele ε4 of the apolipoprotein gene E (*APOE*), which bears the risks of Alzheimer’s disease (whereas allele ε2 possesses protective properties [63, 64]), is associated with increased connectivity in the hippocampus and cortex [65, 66], although this information requires validation [67]. He et al. [68] performed differential analysis of gene coexpression using a biclustering method and have found that 38 genes show various patterns of coexpression between the DMN regions associated and not with Alzheimer’s disease. Functional clusterization showed the involvement of signaling pathways ERBB-4 (ErbB-2 receptor tyrosine kinase 4), ERBB-2 (ErbB-2 receptor tyrosine kinase 2), PTK6 (protein tyrosine kinase 6), non-receptor tyrosine kinase, long-term potentiation, activation of NMDA receptors and related postsynaptic changes, neurotransmitter receptors, and postsynaptic signaling pathways.

A similar approach was applied in the longitudinal study of children, victims of military action [69]. It was shown that the cumulative genetic risk, estimated by the contribution of five alleles, associated with a high risk of development of post-traumatic stress disorder (PTSD), of which three SNPs were variants of the oxytocin receptor gene, determined the dynamics of DMN development with age.

Foo et al. [26] investigated the connection of a wide number of metrics from the graphotheoretical approach and genetic markers in the context of insomnia and functional brain changes related to aging. The authors have found low heritability of graph metrics (h2=0.12), which conflicted with previous studies [70], however, they managed to identified important associations of several SNP (rs62158160, rs62158161, rs62158166, rs62158168, rs12616641, rs62158169, rs62158170, rs199993536, rs6737318, rs62158206, rs7556815, rs2863957, rs1823125, rs60873293), located in the intergenic region in the vicinity of *PAX8* gene (paired box gene 8), with connectivity of somatosensory and limbic networks. The results have demonstrated that insomnia is most significantly related to the decreased connectivity of somatomotor network.

A family of *PAX* genes is known to encode the transcription factors necessary for development and tissue homeostasis [71]. For example, the PAX8 protein is considered a master regulator of cellular processes in DNA repair, replication, and metabolism [72]. It regulates several genes engaged in the production of thyroid hormones [73] needed for the development and functioning of the brain (neuron differentiation, synaptogenesis, and dendrite proliferation) [74, 75].

In the same study [26], the authors showed associations of connectivity strength in the combined network, including 7 networks, with SNP (rs145868127, rs2680724, rs62165320, rs2661030, rs2661035, rs1880544, rs2089478, rs123837764, rs12474078). Additionally, to several non-encoding sequences, five genes have been detected, which were associated with the metrics of global efficiency, specific pathway length, and connectivity strength of DMN, dorsal attention network, and somatosensory network: *SLC25A33* (solute carrier family 25 member 33), *TMEM201* (transmembrane protein 201), *ZEB1* (zinc finger E-box binding homeobox 1), *SH2B3* (SH2B adaptor protein 3), and *ATXN2* (ataxin-2).

As to the genetic markers of functional connectivity patterns in affective disorders, polymorphism of the serotonin transporter gene *5-HTTLPR* (serotonintransporter- linked promoter region) is the most actively studied. Investigations with fMRI [76, 77] have shown that this polymorphism is associated with changes in functional connectivity between the regions, which are responsible for regulation of emotions (amygdala, prefrontal cortex, anterior cingulate gyrus, insular cortex). S-allele 5-HTTLPR has been found to predispose individuals to depression if stressful events occurred in early childhood [78], and also to a number of other mental disorders [79, 80]. It was shown in EEG investigations that in contrast to L-homozigotes, carriers of S-allele have lower density of cortical sources distribution and connectivity in the majority of frequency ranges in the regions overlapping DMN and regions related to emotion regulation [81]. Despite the existence of some papers, in which the link between 5-HTTLPR and stress was not confirmed [82, 83], it is necessary to continue researches, which would consider not only brain connectivity (as an intermediate link) but other factors as well, for example, intricate interaction of genes of serotoninergic system, additional environmental factors, and psychological features. Moreover, it has been found that 5-HTTLPR polymorphism may be related not to the depression as such (in the presence of stressful factors) but rather to the adaptive or non-adaptive strategies of emotion regulation [84].

The study [85], which covered the majority of candidate genes associated with depression and schizophrenia, has shown that when graph analysis method is used, the gene networks in these diseases will have similar properties. It was seen by the number of connections for each node (degree value). The authors stated that the obtained results may partially explain frequent comorbidity of these conditions.

Richiardi et al. [86] estimated non-randomness of gene co-expression in functionally connected brain regions and defined that a pool of expression patterns, in compliance with the principle of belonging to a neural network, determines the graph with a higher degree of connectivity. At the same time, the idea that the coincidence of expression patterns depends on spatial neighborhood of the analyzed regions was excluded. At the stage of biological validation, the association was shown not only with the level of expression but with gene polymorphism as well. Functional annotation demonstrated the significance of the ion channel genes including, for example, GABA5 in determining network connectivity, which confirms the significance of neuron-specific synaptic processes in the development of functional connectivity in the brain.

Summarizing the results of investigations, we can conclude that the link between genetic associations and functional connectivity of the brain is traced in various pathologies. Some current studies are presented in the Table.

**Table:** Investigations of genetic associations with functional human brain connectivity in the context of mental and neurological disorders and predisposition to them

Sample	Genetic associations	Method	Connectivity specifics	Sample size	References
***Association with schizophrenia***					
Patients with schizophrenia and control group	Polymorphism of *nrgn* gene (rs12807809)	Resting-state fMRI	Increase of FC between hippocampus and inferior temporal gyrus, lingual gyrus, and fusiform gyrus in schizophrenia Decrease of FC between hippocampus and caudate nucleus, thalamus, and anterior cingulate gyrus in schizophrenia The lowest FC between hippocampus and anterior cingulate gyrus was found in TT-homozygotes in schizophrenia compared to that in carriers of C-allele in schizophrenia and control group	n=99 (control) and n=59 (schizophrenia): 29 carriers of C-allele and 30 carriers of T-allele	Zhang et al., 2019 [51]
Healthy participants	Genome-wide association study; there were identified 125 genes whose expression was highly significantly associated with connectivity, and 51 genes were associated with schizophrenia	Resting-state fMRI	51 genes associated with schizophrenia turned out to be common for all functional networks and connected with action potentials	n=800 (330 males, 470 females); age — 23.8±2.4 years (18– 30 years)	Zhang et al., 2021 [42]
Healthy participants	COMT (catechol-O-methyltransferase) polymorphism is associated with schizophrenia	fMRI for the task on associative memory	Connection between medial temporal lobe and prefrontal cortex was weaker in COMT val-homozygotes relative to met-homozygotes	11 participants homozygous for COMT val-allele, and 11 participants homozygous for COMT met-allele	Dennis et al., 2010 [55]
***Neurodegenerative changes***					
Patients with Alzheimer’s disease, control group (data from 16 studies)	There were detected 38 genes, which were different in the character of coexpression between brain regions associated and not associated with Alzheimer’s disease in the DMN network	Data of resting-state fMRI from Neurosynth database	Connectivity of DMN was investigated to search for a subset of genes with altered character of coexpression	Total number of participants is not indicated	He et al., 2022 [68]; data of entire brain transcriptomics from the Allen Human Brain Atlas (AHBA)
Middle-aged and elderly participants	One significant locus was identified near PAX8 gene (associated with sleep duration, differentiation and development of neurons, oncology, and susceptibility to neurodegenerative diseases)	Data from resting-state fMRI	Association with connectivity of somatomotor and limbic networks	n=18,445 (9773 females and 8672 males); average age — 62.47± 7.47 years (44– 80 years)	Foo et al., 2021 [26]; Genome-wide data from UK Biobank
***Post-traumatic stress disorder***					
Adolescents — victims of military action and control group	Polymorphisms of genes related to oxytocin: OXTR (rs1042778, rs2254298, rs53576), CD38 — rs3796863, AVPR1A — RS3	Resting-state MEG	Total contribution of 5 polymorphisms and symptoms related to stress explained 25% of differences in DMN connectivity	n=74 (age — 11– 13 years), 39 of them are victims of military action, 35 — control group	Zeev-Wolf et al., 2020 [69]
***Affective disorders***					
Healthy participants	5-HTTLPR polymorphism (associated with depression an a number of mental disorders)	fMRI during perception of emotional expression of faces	Lower connectivity between amygdale and anterior cingulate gyrus was observed in carriers of S-allele than in carriers of L-homozygotes	n=94 (age and gender are not indicated)	Pezawas et al., 2005 [87]
Healthy participants	5-HTTLPR polymorphism	Resting-state EEG	Lower density of distribution of cortical EEG signal sources and connectivity in the majority of frequency ranges in the regions overlapping DMN and regions related to emotion regulation was observed in S-allele homo-and heterozygotes in comparison with L-allele homozygotes	n=113 (63% are females, average age — 25.2±8.9)	Proshina et al., 2018 [81]

Note: FC — functional connectivity, MEG — magnetoencephalography.

It is clear that the condition of the intercellular matrix, endothelial activity, and other factors determining anatomical connectivity and structuring of the grey and white matter influence specific neuron activity and efficiency of synaptic connections. Age and other epigenetic factors make their contribution to the formation of connectome variability [88]. Additionally to the factors of structural connectivity, the geometry, cytoarchitecture, and patterns of gene expression determine functional connectivity to the greatest degree [89, 90]. However, current approaches to the estimation of connectome genetics are a powerful tool enabling discrimination of factors, which impact specifically structural and functional connectivity.

## Conclusion

Data, accumulated by the present time, allow us to believe that functionally clustered connectivity genes refer to the pool of ion channel regulation, calcium homeostasis, neuroplasticity, neurotransmitter release. This is an indication of the priority (essentiality) of neuron-specific synaptic processes in the establishing of functional connectivity in the brain. This information may be applied in the field of translation medicine and clinical practice, since it gives an idea of potential targets of therapeutic action.

This is especially important due to the necessity of developing more advanced methods of early diagnosing and treatment of mental disorders including affective pathologies and neurodegenerative diseases, the growing occurrences of which pose a serious threat for functioning of an individual and society in general. Exploration of close connection of genetic factors with brain functioning in psychopathologies provides the possibility to obtain objective information, which may be used as an option to the standard diagnostic procedures. The search for objective markers is of special importance in view of a large temporal gap between the onset of the disease development and manifestation of clinical symptoms. One of the most important tasks of the current neurosciences is fixation of early cognitive deviations, which may slow down the development of neurodegenerative diseases. In the near future, owing to the development of personalized medicine, a question of the degree of association of connectivity, complexity, and other metrics of functional networks with individual specific genotype will attract increasing interest.
